# P-1760. Pre- and Post-Guideline Implementation Comparison of Skin and Soft Tissue Infection Empiric Antibiotic Prescribing and Antimicrobial Stewardship Interventions

**DOI:** 10.1093/ofid/ofae631.1923

**Published:** 2025-01-29

**Authors:** Mitchel Ryan, Brady A Caverzagie, Jennifer Ross, Aditya Chandorkar

**Affiliations:** M Health Fairview, Shoreview, Minnesota; M Health Fairview University of Minnesota Medical Center, Minneapolis, Minnesota; M Health Fairview - University of Minnesota Medical Center, Minneapolis, Minnesota; University of Minnesota, Minneapolis, Minnesota

## Abstract

**Background:**

Skin and soft tissue infections (SSTIs) are common in the acute care setting, ranging from uncomplicated cellulitis to necrotizing fasciitis. In 2021, a retrospective review was completed in patients admitted from the Emergency Department (ED) with an SSTI to Lakes Medical Center, a small community hospital, to assess prescribing habits and antimicrobial stewardship (AMS) interventions. An evidence-based guideline for empiric treatment of SSTIs in adults was introduced in 2022 to establish a best practice framework. The purpose of this study is to assess changes in empiric antibiotic use with SSTIs and evaluate guideline adherence following implementation.

Antibiotic Breakdown

**Figure:**
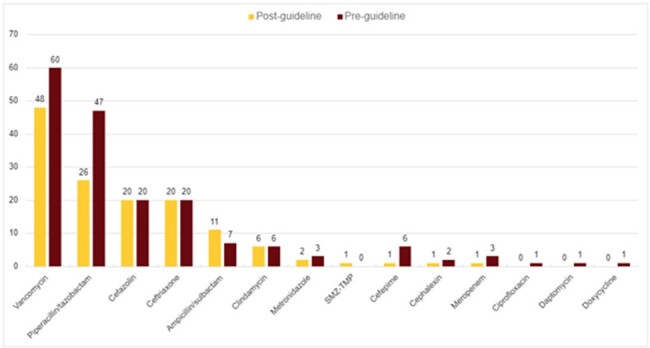

**Methods:**

Adult patients started on antibiotics in the ED for an SSTI and subsequently admitted between 1/2023 and 12/2023 were included in this retrospective pre/post implementation study. Patients diagnosed with an SSTI after admission or not started on empiric antibiotics in the ED were excluded. The type of SSTI was determined by ED provider description and pictures uploaded to the electronic health record. Allergies to antibiotics, previous therapies, MRSA history and SIRS criteria were assessed upon presentation. A Fisher Exact test was used to determine significance between categorical values.

**Figure:**
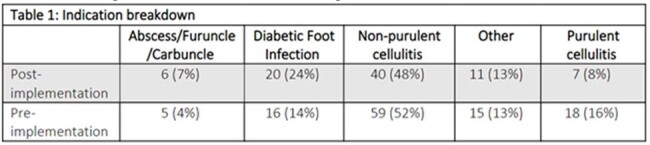

**Results:**

Eighty-four patients were included in the post-intervention group compared to 113 patients in the pre-implementation cohort. The proportion of patients by indication were similar between pre/post implementation patients (Table 1). There was no significant difference in the number of patients diagnosed with a non-purulent cellulitis (p= .57). A significant difference was seen in antipseudomonal antimicrobials (p=.029) (Figure 1). Combination therapy was initiated in a similar proportion of patients (p= 1) (Table 2). Pharmacist driven AMS interventions decreased following guideline implementation from 11 to 6 among non-purulent cellulitis cases (p= .43) (Table 3).

**Figure:**
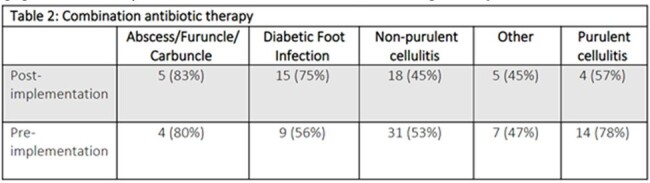

**Conclusion:**

Following the implementation of an SSTI guideline, there were significantly fewer patients started on empiric anti-pseudomonal antimicrobials for SSTIs. There was a trend towards decreased antimicrobial stewardship interventions post-implementation.

**Figure:**
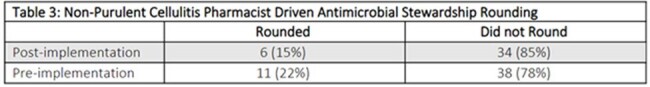

**Disclosures:**

**Jennifer Ross, PharmD, BCIDP**, Shionogi Inc.: Advisor/Consultant

